# Supplementary material for: Untranslated Region Sequences and the Efficacy of mRNA Vaccines against Tuberculosis
Source: Int J Mol Sci. 2024 Jan 10;25(2):888. doi: 10.3390/ijms25020888 (PMC10815675; doi:10.3390/ijms25020888)
Supplement: Supplementary file 1 [file ijms-25-00888-s001.zip › Supplement Figure S1 R1.pdf]

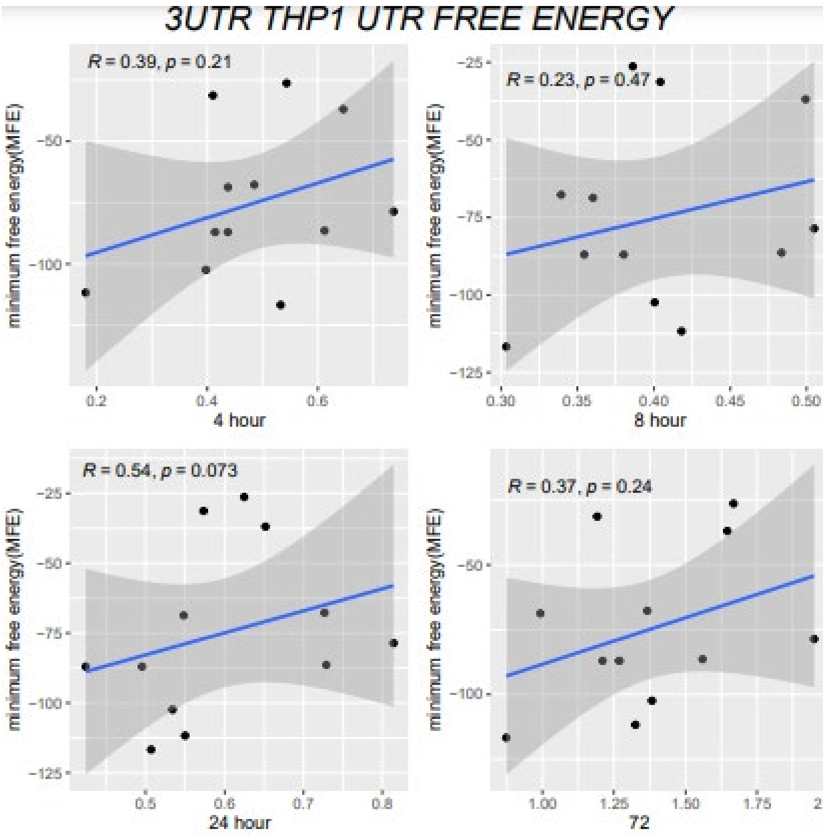
3UTR THP1 UTR FREE ENERGY
R = 0.39, p = 0.21
R = 0.23, p = 0.47
R = 0.54, p = 0.073
R = 0.37, p = 0.24
minimum free energy(MFE)
4 hour
8 hour
24 hour
72

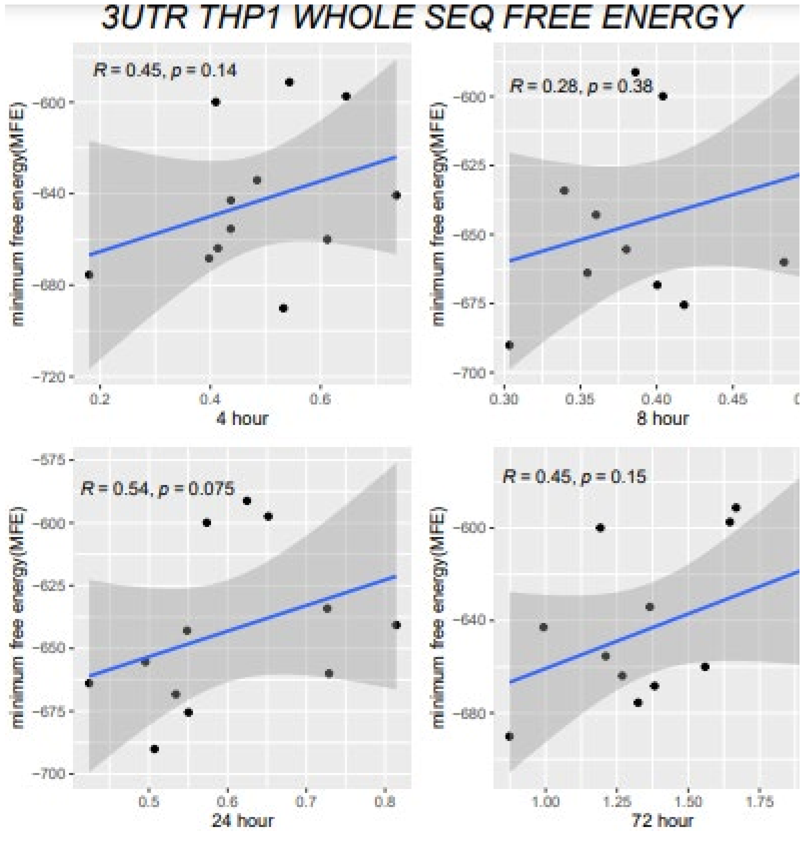
3UTR THP1 WHOLE SEQ FREE ENERGY
R = 0.45, p = 0.14
R = 0.28, p = 0.38
R = 0.54, p = 0.075
R = 0.45, p = 0.15
minimum free energy(MFE)
4 hour
8 hour
24 hour
72 hour

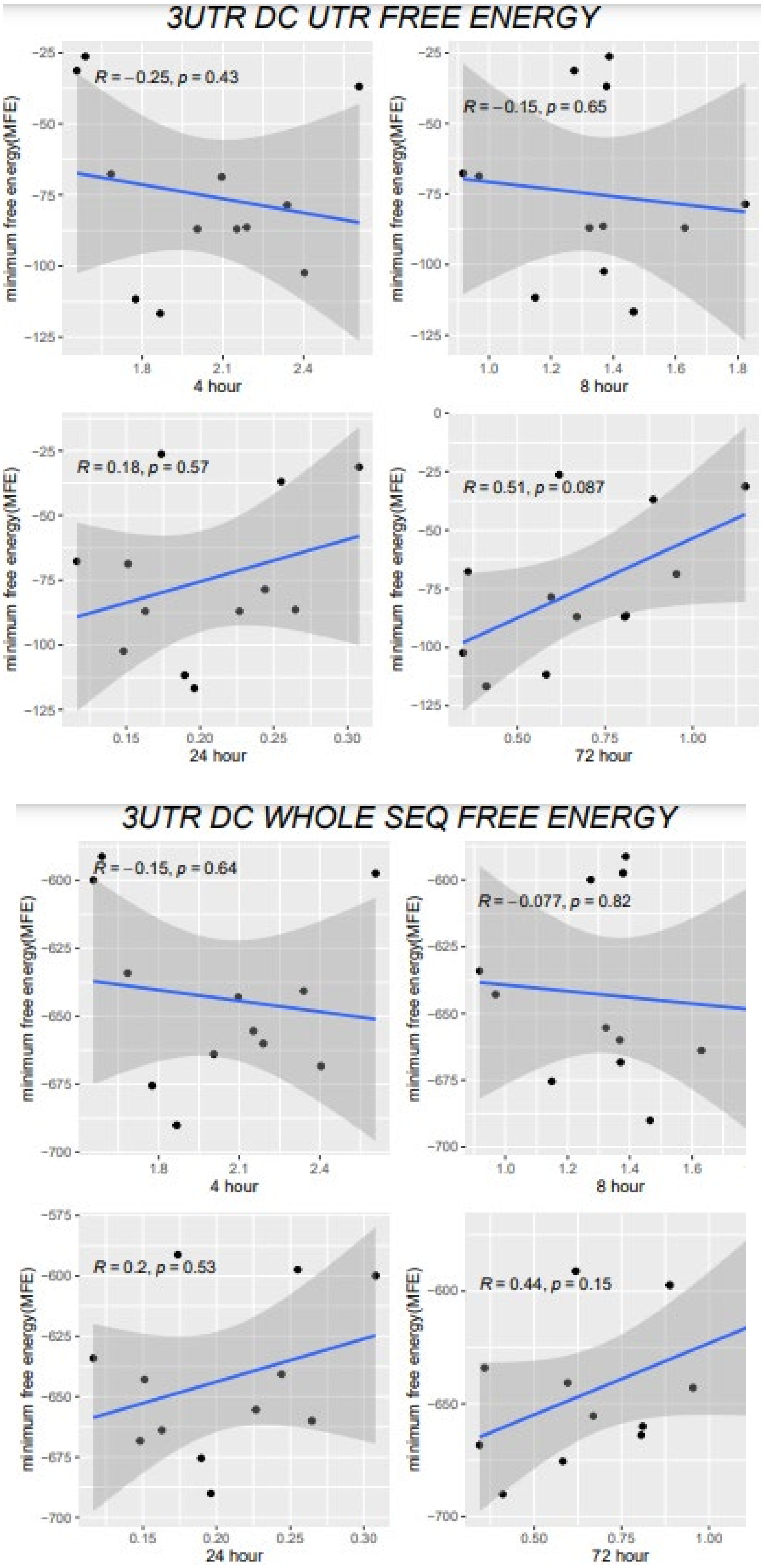


**Supplementary Figure S1.** Correlation between minimum free energy (MFE) RNA sequences and intensity of bioluminescence.
