# Supplementary material for: Untranslated Region Sequences and the Efficacy of mRNA Vaccines against Tuberculosis
Source: Int J Mol Sci. 2024 Jan 10;25(2):888. doi: 10.3390/ijms25020888 (PMC10815675; doi:10.3390/ijms25020888)
Supplement: Supplementary file 1 [file ijms-25-00888-s001.zip › Supplement Table S2 R1.pdf]

Supplementary Table S2. 5′ and 3′UTRs sequences.

Name	UTR	Sequence
FH1	5'UTR	GGGAAATAGCCCAGAAATTCTACCCAAGCTCCCTCGCCAC
GAPDH	5'UTR	GGGAAATAGCTCTCTGCTCCTCCTGTTCGACAGTCAGCCGCATCTTCTT TTGCGTCGCCAGCCGAGCCACATCGCTCAGCCACC
eEF2	5'UTR	GGGAAATACACATCCGCCGTCGTCGCCGCCATCCTCGGCGCGACTCGC TTCTTTCGGTTCTACCTGGGAGAATCCACCGCCATCCGCCACC
HSPA1A	5'UTR	GGGAAATAACGGCTAGCCTGAGGAGCTGCTGCGACAGTCCACTACCT TTTTCGAGAGTGACTCCCGTTGTCCCAAGGCTTCCCAGAGCGAACCTG TGCGGCTGCAGGCACCGGCGCGTCGAGTTTCCGGCGTCCGGAAGGAC CGAGCTCTTCTCGCGGATCCAGTGTTCCGTTTCCAGCCCCCAATCTCAG AGCGGAGCCGACAGAGAGCAGGGAACCGGCGCCACC
H4C2	5'UTR	GGGAAATAGTACCTGGTTTTTGTTGTCAGCTGGTTAGCCACC
HBB	5'UTR	GGGAAATACATTTGCTTCTGACACAACTGTGTTCACTAGCAACCTCAA ACAGCCACC
C3	5'UTR	GGGAAATACTCCTCCCCATCCTCTCCCTCTGTCCCTCTGTCCCTCTGAC CCTGCACTGTCCCAGCCACC
CFL1	5'UTR	GGGAAATAGTTCAGTCGGGTCCCGGCAGCGGCTGCAGCGCTCTCGTCT TCTGCGGCTCTCGGTGCCCTCTCCTTTTCGTTTCCGCCACC
Rabb	5'UTR	GGGAAATACACTTGCTTTGACACAACTGTGTTTACTTGCAATCCCCCA AAACAGACAGACGCCACC
TPL	5'UTR	GGGAAATACTCTCTTCGCATCGCTGTCTGCGAGGGCCAGCTGTTGGGC TCGCGGTTGAGGACAAACTCTTCGCGGTCTTTCCAGTACTCTTGGATC GGAAACCCGTCGGCCTCCGAACGGTACTCCGCCACCGAGGGACCTGA GCGAGTCCGCATCGACCGGATCGGAAAACCTCTCGAGAAAGGCGTCT AACCAGTCACAGTCGCAAGAAGCTTAGCATTCCGGTAACTGTTGGTAA AGCCACC
RNA-1273 (Moderna)	5'UTR	GGGAAATAAGAGAGAAAAGAAGAGTAAGAAGAAATATAAGACCCCG GCGCCGCCACC
BNT162b2 (Bionthech)	5'UTR	GAGAATAAACTTGTATTCTTCTGGTCCCCACAGACTCAGAGAGAACCC GCCACC
HBB	3'UTR	GATCCTGGACTGTTTCCTGATAACCATAAGAAGACCCTATTTCCCTAG ATTCTATTTTCTGAACTTGGGAACACAATGCCTACTTCAAGGGTATGG CTTCTGCCTAATAAAGAATGTTCAGCTCAA
HBB-EMCV	3'UTR	GATCCTGGACTGTTTCCTGATAACCATAAGAAGACCCTATTTCCCTAG ATTCTATTTTCTGAACTTGGGAACACAATGCCTACTTCAAGGGTATGG CTTCTGCCTAATAAAGAATGTTCAGCTCAAGGGGCTGAAGGATGCCCA GAAGGTACCCCATTGTATGGGATCTGATCTGGGGCCTCGGTGCACATG CTTTACATGTGTTTAGTCGAGGTTAAAAAGCGTCTAGGCCCCCCGAAC CACGGGGACGTGGTTTTCCTTTGAAAAACACGATGATAATATGGCCAC AACCGGCTTCTGCCTAATAAAGAATGTTCAGCTCAA
mtRNR-EMCV	3'UTR	CAAGCACGCAGCAATGCAGCTCAAAACGCTTAGCCTAGCCACACCCC CACGGGAAACAGCAGTGATTAACCTTTGCAATAAACGAAAGTTTAAC TAAGCTATACTAACCCCAGGGTTGGTCAATTTCGTGCCAGCCACACCC TGGAGCTAGCGGGGCTGAAGGATGCCCAGAAGGTACCCCATTGTATG GGATCTGATCTGGGGCCTCGGTGCACATGCTTTACATGTGTTTAGTCG AGGTTAAAAAGCGTCTAGGCCCCCCGAACCACGGGGACGTGGTTTTCC TTTGAAAAACACGATGATAATATGGCCACAACCGGCTTCTGCCTAATA AAGAATGTTCAGCTCAA
mtRNR	3'UTR	CAAGCACGCAGCAATGCAGCTCAAAACGCTTAGCCTAGCCACACCCC CACGGGAAACAGCAGTGATTAACCTTTGCAATAAACGAAAGTTTAAC

		TAAGCTATACTAACCCCAGGGTTGGTCAATTTCGTGCCAGCCACACCC<br>TGGAGCTAGC
mtRNR-<br>mtRNR	3'UTR	CAAGCACGCAGCAATGCAGCTCAAAACGCTTAGCCTAGCCACACCCC<br>CACGGGAAACAGCAGTGATTAACCTTTGCAATAAACGAAAGTTTAAC<br>TAAGCTATACTAACCCCAGGGTTGGTCAATTTCGTGCCAGCCACACCC<br>TGGAGCTAGCCAAGCACGCAGCAATGCAGCTCAAAACGCTTAGCCTA<br>GCCACACCCCCACGGGAAACAGCAGTGATTAACCTTTGCAATAAACG<br>AAAGTTTAACTAAGCTATACTAACCCCAGGGTTGGTCAATTTCGTGCC<br>AGCCACACCCTGGAGCTAGC
mtRNR-<br>AES	3'UTR	CAAGCACGCAGCAATGCAGCTCAAAACGCTTAGCCTAGCCACACCCC<br>CACGGGAAACAGCAGTGATTAACCTTTGCAATAAACGAAAGTTTAAC<br>TAAGCTATACTAACCCCAGGGTTGGTCAATTTCGTGCCAGCCACACCC<br>TGGAGCTAGCCTCGAGCTGGTACTGCATGCACGCAATGCTAGCTGCCC<br>CTTTCCCGTCCTGGGTACCCCGAGTCTCCCCCGACCTCGGGTCCCAGG<br>TATGCTCCCACCTCCACCTGCCCCACTCACCACCTCTGCTAGTTCCAGA<br>CACCTCC
mtRNR-<br>HBB	3'UTR	CAAGCACGCAGCAATGCAGCTCAAAACGCTTAGCCTAGCCACACCCC<br>CACGGGAAACAGCAGTGATTAACCTTTGCAATAAACGAAAGTTTAAC<br>TAAGCTATACTAACCCCAGGGTTGGTCAATTTCGTGCCAGCCACACCC<br>TGGAGCTAGCGATCCTGGACTGTTTCCTGATAACCATAAGAAGACCCT<br>ATTTCCCTAGATTCTATTTTCTGAACTTGGGAACACAATGCCTACTTCA<br>AGGGTATGGCTTCTGCCTAATAAAGAATGTTCAGCTCAA
AES-HBB	3'UTR	CTCGAGCTGGTACTGCATGCACGCAATGCTAGCTGCCCCTTTCCCGTC<br>CTGGGTACCCCGAGTCTCCCCCGACCTCGGGTCCCAGGTATGCTCCCA<br>CCTCCACCTGCCCCACTCACCACCTCTGCTAGTTCCAGACACCTCCGAT<br>CCTGGACTGTTTCCTGATAACCATAAGAAGACCCTATTTCCCTAGATT<br>CTATTTTCTGAACTTGGGAACACAATGCCTACTTCAAGGGTATGGCTT<br>CTGCCTAATAAAGAATGTTCAGCTCAA
AES-EMCV	3'UTR	CTCGAGCTGGTACTGCATGCACGCAATGCTAGCTGCCCCTTTCCCGTC<br>CTGGGTACCCCGAGTCTCCCCCGACCTCGGGTCCCAGGTATGCTCCCA<br>CCTCCACCTGCCCCACTCACCACCTCTGCTAGTTCCAGACACCTCCGG<br>GGCTGAAGGATGCCCAGAAGGTACCCCATTGTATGGGATCTGATCTGG<br>GGCCTCGGTGCACATGCTTTACATGTGTTTAGTCGAGGTTAAAAAGCG<br>TCTAGGCCCCCCGAACCACGGGGACGTGGTTTTCCTTTGAAAAACACG<br>ATGATAATATGGCCACAACCGGCTTCTGCCTAATAAAGAATGTTCAGC<br>TCAA
AES-AES	3'UTR	CTCGAGCTGGTACTGCATGCACGCAATGCTAGCTGCCCCTTTCCCGTC<br>CTGGGTACCCCGAGTCTCCCCCGACCTCGGGTCCCAGGTATGCTCCCA<br>CCTCCACCTGCCCCACTCACCACCTCTGCTAGTTCCAGACACCTCCCTC<br>GAGCTGGTACTGCATGCACGCAATGCTAGCTGCCCCTTTCCCGTCCTG<br>GGTACCCCGAGTCTCCCCCGACCTCGGGTCCCAGGTATGCTCCCACCT<br>CCACCTGCCCCACTCACCACCTCTGCTAGTTCCAGACACCTCC
RNA-1273<br>(Moderna)	3'UTR	GCTGGAGCCTCGGTGGCCTAGCTTCTTGCCCCTTGGGCCTCCCCCCAG<br>CCCCTCCTCCCCTTCCTGCACCGTACCCCCGTGTCTTTGAATAAAGTCT<br>GAGTGGGCGGCA
BNT162b2<br>(Bionthech)	3'UTR	CTCGAGCTGGTACTGCATGCACGCAATGCTAGCTGCCCCTTTCCCGTC<br>CTGGGTACCCCGAGTCTCCCCCGACCTCGGGTCCCAGGTATGCTCCCA<br>CCTCCACCTGCCCCACTCACCACCTCTGCTAGTTCCAGACACCTCCCA<br>AGCACGCAGCAATGCAGCTCAAAACGCTTAGCCTAGCCACACCCCCA<br>CGGGAAACAGCAGTGATTAACCTTTGCAATAAACGAAAGTTTAACTA<br>AGCTATACTAACCCCAGGGTTGGTCAATTTCGTGCCAGCCACACCCTG<br>GAGCTAGC
